# The risk of child and adolescent overweight is related to types of food consumed

**DOI:** 10.1186/1475-2891-10-71

**Published:** 2011-06-24

**Authors:** Vichuda L Matthews, Michelle Wien, Joan Sabaté

**Affiliations:** 1 Department of Epidemiology and Biostatistics, School of Public Health, Loma Linda University, Loma Linda, California, USA; 2 Department of Nutrition, School of Public Health, Loma Linda University, Loma Linda, California, USA

## Abstract

**Background/Aims:**

To investigate the association between the risk of overweight and the consumption of food groups in children and adolescents.

**Methods:**

We studied 1764 healthy children and adolescents (age 6-19y) attending 16 Seventh-Day Adventist schools and 13 public schools using a 106-item non-quantitative food frequency questionnaire from the late 1980 Child-Adolescent Blood Pressure Study. Logistic regression models were used to compute the risk of overweight according to consumption of grains, nuts, vegetables, fruits, meats/fish/eggs, dairy, and, low nutrient-dense foods (LNDF).

**Results:**

The frequency of consumption of grains, nuts, vegetables and LNDF were inversely related to the risk of being overweight and dairy increased the risk. Specifically, the odds ratio (95% CI) for children in the highest quartile or tertile of consumption compared with the lowest quartile or tertile were as follows: grains 0.59(0.41-0.83); nuts 0.60(0.43-0.85); vegetables 0.67(0.48-0.94); LNDF 0.43(0.29-0.63); and, dairy 1.36(0.97, 1.92).

**Conclusion:**

The regular intake of specific plant foods may prevent overweight among children and adolescents.

## Introduction

The global burden of obesity among children and adolescents is estimated to be 1 in 10,[1] which has profound public health implications in the context of chronic disease prevention and lifespan. It has been determined from National Health and Nutrition Examination Survey (NHANES) data gathered between 2007 and 2008 that the prevalence of children and adolescents between the age of 2 to 19 years that were at or above the 85^th ^percentile of age specified BMI values in the United States (US) were almost 32%, hence one in three children is either overweight or obese [2].

Dietary intake and physical activity are the cornerstones of weight management across the life cycle and these factors play an important role in influencing the likelihood of being overweight during childhood and adolescence. In addition to the decline in physical activity observed during adolescence [3] and the known association of hours of TV watching with childhood overweight [4-6], it is plausible that the current child and adolescent overweight epidemic is a consequence of excessive or inadequate consumption of specific food groups. Also, Jahns et al found an increased prevalence of snacking among children in the US from 1977 to 1996 [7]. More specifically, there was an increase in energy density and proportion of energy from fat and decrease in calcium density obtained from snacks. Findings from NHANES have consistently reported an insufficient consumption of fruits and vegetables among children and adolescents in the US [8,9]. Additionally, studies performed in the 1970s and 1980s have shown that vegetarian children tend to be lighter and leaner than non-vegetarian children [10-14].

The balance between long-term energy intake versus energy expenditure contributes to the likelihood of being overweight or obese. Therefore, plant-based food groups that are inherently high in fiber and low in fat are traditionally recommended to prevent obesity. The type of foods in the diet may influence a child or adolescent's body mass index (BMI), which serves as a surrogate measure for classifying overweight and obese conditions. In light of the increasing prevalence of child and adolescent overweight and obesity worldwide, there is a critical need to identify nutrition related-risk factors by evaluating the intake of food groups and anthropometric data from large scale studies involving children and adolescents. Therefore, the purpose of this study is to examine the association between overweight and the intake of several food groups by children and adolescents who participated in the Child-Adolescent Blood Pressure Study, a large epidemiological study [15].

## Subjects and Methods

The study design and data collection procedures for the Child-Adolescent Blood Pressure Study have been described previously [15-17]. In brief, height and weight measurements were taken from 1764 children and adolescents attending first through 12^th ^grade at 16 Seventh-Day Adventist (Adventist) schools and 13 public schools in Southern California. This project was approved by the Institutional Review Board of Loma Linda University. Informed consent was obtained from the parents before initiation of data collection. Height was recorded to the nearest ¼ inch by two observers using a portable stadiometer with the subject standing erect in stocking feet without upward pressure exerted on the mastoids. Weight was recorded without shoes using a portable beam scale by two observers. BMI was calculated as weight in kilograms divided by height in meters squared.

Dietary information was obtained from 870 Adventist and 894 public school students using a validated 106-item non-quantitative food frequency questionnaire (FFQ) [17]. The FFQ was developed [18] and pretested (unpublished data) in 1978 on 211 schoolchildren aged 9 to 18 years. Frequency choices were never, every month, every week and every day and were assigned the frequency values of 0, 1, 4 and 30 per month, respectively. The questionnaire did not include questions regarding the number of hours of daily TV watching or amount of time engaged in daily physical activity. The FFQ was self-administered by all students aged 10 years or older and completed by the mothers of students aged 6 to 9 years. All of the foods with the exception of diet soda and three non-conforming foods were classified into seven food groups by a Registered Dietitian as follows: grains (e.g. cereals, crackers, bread, biscuit, muffin, pancakes, French toast, waffles, macaroni, noodles, spaghetti, meat substitute products, granola bar, pop tart), nuts (nuts and peanut butter), fruits (e.g. apple, citrus, banana, other fruits, canned fruits, raisins, dried fruits, orange juice, other fruit juice), vegetables (e.g. vegetable salad, carrot sticks, celery sticks, potatoes, green beans, refried beans, other beans, cooked vegetable), dairy (e.g. whole milk, chocolate milk, cottage cheese, cheeses, yogurt, pudding, ice cream, frozen yogurt, milk shake), meats (e.g. eggs, real wieners, hamburger patty, steak, roast beef, fried chicken, chicken, bacon, sausage links, bologna, ham, pork chops, fish) and low nutrient-dense foods (LNDF) (e.g. punch, donuts, sweet roll, chips, French fries, other fried potatoes, candy bars, other candy bar, cookies, cake, pie, twinkie, popsicle). Combination of meat and grain foods (e.g. burrito, taco, pizza, tostada, hamburger on bun) were counted as half serving of meat and half serving of grain. Combination of meat and vegetable foods (e.g. soup, stew, chili) were counted as half serving of meat and half serving of vegetable. Combination of LDNF and dairy foods (e.g. ice cream bar) were counted as half serving of LDNF and half serving of dairy. Combination of LDNF and grain foods (e.g. ice cream cone, frozen yogurt cone) were counted as half serving of LDNF and half serving of grain.

For each student, the frequency of consumption for each of the aforementioned food groups was computed and ranked using quartiles for analytical purposes. Due to a narrow range of consumption, tertiles were used for nuts. The age and gender specific 85^th ^percentile for BMI of the study population was used as a cut-off point to identify children and adolescents as being overweight according to current recommendations [19]. The students were a relatively healthy population residing within health-oriented Southern California communities with a low prevalence of obesity.

## Statistical Analysis

Data was evaluated using Statistical Analysis Systems software version 9.1 (SAS, Cary, North Carolina). Logistic regression was performed using two models to determine the association between overweight (>85^th ^percentile) and frequency of consumption for the seven food groups. In light of a potential association between soda intake and risk of overweight, the absolute and relative frequency of consumption of soda intake was evaluated. The absolute and relative frequency of soda intake comparing the lowest quartile (Q1) to the highest quartile (Q4) showed a monotonic dose response with the risk of overweight; hence soda intake was included in the two models. Odds ratios (OR) and 95% confidence intervals (CI) were calculated from two separate models. Model 1 included overweight as the dependent variable and frequency of consumption of a food group was the exposure variable controlling for gender (boys vs. girls), type of school (Adventist vs. public) and soda intake. Adjustment on the type of school was based on the previous findings where there was a difference in weight among students enrolled in the Adventist and public school systems [16]. Model 2 was the same as Model 1 but included additional simultaneous adjustments for the frequency of consumption of all of the other six food groups.

## Results

In boys, the average BMI increased with age with the exception of the strata ages 9.5 to 10.4 years (n = 47) (Table 1). In girls, the average BMI increased with age with the exception of the strata ages 9.5 to 10.4 (n = 44) and 11.5 to 12.4 (n = 100). The total number of overweight cases for boys and girls was 151 (17%) and 176 (20%), respectively. Among boys, the 85^th ^percentile for BMI values peaked at the age of 8.5 to 9.4 years and 16.5 to 17.4 years. Among girls, the 85^th ^percentile for BMI values peaked at the age of 10.5 to 11.4 years, 14.5 to 15.4 years and 16.5 to 17.4 years. The basic logistic regression model showed null findings for age, gender and school in the context of >85^th ^percentile for BMI. Further statistical adjustment for parent's body weight did not change the study findings.

**Table 1:** Age and gender specific BMI of students with completed dietary questionnaires (n = 1764)

Age (yr)	n	Mean ±SD for BMI	85^th ^Percentile for BMI	Overweight cases
**Boys**				
7.5-8.4	18	15.58 ± 1.78	17.50	4
8.5-9.4	34	17.36 ± 1.64	19.30	13
9.5-10.4	47	16.67 ± 1.73	17.90	8
10.5-11.4	72	17.65 ± 2.77	20.26	11
11.5-12.4	88	18.19 ± 2.62	20.34	14
12.5-13.4	107	18.65 ± 3.07	22.00	17
13.5-14.4	121	19.58 ± 3.92	22.20	19
14.5-15.4	125	20.01 ± 3.03	22.30	19
15.5-16.4	127	20.64 ± 2.24	22.80	20
16.5-17.4	99	21.72 ± 3.22	24.50	19
17.5-18.4	41	21.74 ± 2.82	23.70	7
**Girls**				
7.5-8.4	24	15.66 ± 1.45	16.90	4
8.5-9.4	49	16.88 ± 2.53	18.70	8
9.5-10.4	44	16.68 ± 1.93	18.98	7
10.5-11.4	74	17.92 ± 2.86	20.60	15
11.5-12.4	100	17.85 ± 2.50	19.96	16
12.5-13.4	83	18.82 ± 2.88	21.30	13
13.5-14.4	115	19.95 ± 3.51	22.40	18
14.5-15.4	154	20.36 ± 3.10	23.30	35
15.5-16.4	128	20.75 ± 3.23	23.00	32
16.5-17.4	86	20.83 ± 2.54	23.24	23
17.5-18.4	28	21.65 ± 4.69	22.00	5

A wide range in the median frequency of intake for the lowest quartile (Q1) or tertile (T1) versus highest quartile (Q4) or tertile (T3) was found for all the food groups (Table 2). The largest differences observed were 3.8 servings/day for fruits and 2.7 servings/day for grains, whereas the smallest differences were 1.0 servings/day for nuts and 1.7 servings/day for dairy.

**Table 2:** Risk of overweight according to frequency of consumption of food groups

Quartile (Q) or Tertile (T)	median servings/d	**Model 1**^**a**^	**Model 2**^**b**^
		OR (95%CI)	OR (95%CI)
**Grains**			
Q1	0.8	1.00	1.00
Q2	1.4	**0.69 (0.50, 0.96)**	0.77 (0.54, 1.10)
Q3	2.1	**0.61 (0.44, 0.87)**	0.73 (0.50, 1.07)
Q4	3.5	**0.59 (0.41, 0.83)**	0.78 (0.51, 1.20)
		*P Trend = 0.002*	*P Trend = 0.19*
**Nuts**			
T1	0.1	1.00	1.00
T2	0.2	**0.75 (0.57, 0.98)**	0.81 (0.61, 1.08)
T3	1.1	**0.60 (0.43, 0.85)**	**0.68 (0.46, 0.98)**
		*P Trend = 0.002*	*P Trend = 0.03*
**Vegetables**			
Q1	0.4	1.00	1.00
Q2	0.7	**0.65 (0.46, 0.92)**	0.73 (0.51, 1.06)
Q3	1.5	0.90 (0.65, 1.24)	0.93 (0.66, 1.32)
Q4	2.6	**0.67 (0.48, 0.94)**	0.68 (0.45, 1.03)
		*P Trend = 0.09*	*P Trend = 0.19*
**Fruits**			
Q1	0.4	1.00	1.00
Q2	0.9	0.83 (0.59, 1.15)	0.92 (0.65, 1.32)
Q3	1.8	0.92 (0.66, 1.29)	1.02 (0.71, 1.48)
Q4	4.2	0.78 (0.55, 1.12)	0.96 (0.63, 1.47)
		*P Trend = 0.27*	*P Trend = 0.98*
**Meats/Fish/Eggs**			
Q1	0.3	1.00	1.00
Q2	0.8	1.08 (0.73, 1.58)	1.02 (0.69, 1.53)
Q3	1.3	1.13 (0.76, 1.69)	1.30 (0.85, 1.99)
Q4	2.5	0.83 (0.54, 1.28)	1.06 (0.66, 1.70)
		*P Trend = 0.34*	*P Trend = 0.64*
**Dairy**			
Q1	0.6	1.00	1.00
Q2	1.3	1.07 (0.76, 1.50)	1.15 (0.81, 1.63)
Q3	1.4	1.06 (0.74, 1.51)	1.30 (0.89, 1.91)
Q4	2.3	1.36 (0.97, 1.92)	**1.99 (1.34, 2.94)**
		*P Trend = 0.09*	*P Trend = 0.0008*
**Low Nutrient-Dense Foods**			
Q1	0.4	1.00	1.00
Q2	0.8	0.90 (0.65, 1.25)	0.91 (0.65, 1.29)
Q3	1.5	**0.68 (0.48, 0.96)**	**0.68 (0.47, 0.98)**
Q4	3.0	**0.43 (0.29, 0.63)**	**0.46 (0.30, 0.71)**
		*P Trend < 0.0001*	*P Trend = 0.0001*

^a^Frequency of consumption of a food group controlling for gender, type of school and soda intake (Logistic regression model: Overweight = one food group + gender + type of school + soda consumption).

^b^Same as Model 1 plus additional simultaneous adjustments for frequency of consumption of all the other six food groups. (Logistic regression model: Overweight = Grains + Nuts + Vegetables + Fruits + Meat/Fish/Eggs + Dairy + LNDF + gender + type of school + soda consumption).

The risk (OR) of overweight according to the frequency of consumption of the seven food groups is shown in Table 2, showing the univariate OR of overweight for the highest tertile or quartile as compared to the lowest tertile or quartile (reference group). Compared to students consuming grains in Q1, greater consumption of grains significantly decreased the likelihood of overweight by 31%, 39% and 41% at the Q2, Q3 and Q4 levels, respectively (*P Trend = 0.002*). Nut intake significantly decreased the likelihood of overweight in Model 1 by 25% and 40% at the T2 and T3 levels, respectively (*P Trend *= 0.002). The effect of nuts was slightly attenuated to 32% at the T3 level in Model 2 (*P Trend *= 0.03). Vegetable intake was found to be protective for the risk of overweight in Model 1 at the Q2 and Q4 levels, 35% and 33% respectively (*P Trend *= 0.09).

No significant differences in risk of overweight were found for fruits and meats/fish/eggs in the two models. In Model 2, compared to students consuming dairy at the Q1 level, dairy intake significantly increased the likelihood of overweight at the Q4 level by 99% (*P Trend *= 0.0008). In both models, LNDF intake decreased the likelihood of overweight at the Q3 (32%) and Q4 (54 to 57% range) levels (*P Trend *< 0.001).

## Discussion

This study has shown that specific plant-based food groups may have a protective role in preventing overweight among children and adolescents whereas dairy intake may be associated with an increased risk of overweight. Using two logistic regression analysis models controlling for gender, type of school and soda intake with and without further adjustment for other food groups, we observed a consistent inverse relationship and significant linear trend for the intake of grains, nuts and LNDF and likelihood of overweight. Although fruits and vegetables are plant-based food groups, we only observed a protective effect on the risk of overweight with the intake of vegetables, whereas the fruit group showed null findings.

Several food attributes may contribute to the consistent protective effects shown for grains and the risk of overweight. Grains are nutrient dense in light of their high complex carbohydrate and fiber content which support satiety[20] and leanness. With the exception of ready-to-eat (RTE) cereals, scant attention has been given to evaluating the effect of grain intake on the risk of overweight among children and adolescents. In a study of Greek adolescents, consuming RTE cereal for breakfast was associated with a 0.67 risk of overweight (95% CI: 0.52, 0.98) [21]. Our current findings support those of Newby et al that found a 0.16 kg smaller weight gain per year (*P *< 0.01) with each additional daily serving of grains and breads among preschool children [22].

The present study establishes that an inverse association exists between nut consumption and risk of overweight in children and adolescents, as has been consistently found in previous epidemiological studies and clinical trials in adults[23,24]. Several plausible reasons exist to explain why nut consumption is not associated with increased BMI, including increased resting metabolic rate, enhanced satiety and corresponding decreased intake of other foods, and incomplete absorption of energy from nuts. Alper and Mattes observed up to an 11% greater resting energy expenditure after 19 weeks of peanut supplementation in adults [25]. Although nuts are more energy dense due to their high fat content, they are a good source of protein and are low in saturated fat and trans fat, which have the strongest association with the risk of weight gain [26]. Furthermore, the link between dietary fat intake and obesity is weak and inconsistent in the context of epidemiological studies and long-term randomized trials [26,27]. Foods that contain good sources of fiber and protein have been shown to produce increased satiety ratings [28,29]. More specifically, Kirkmeyer and Mattes [30] found that peanuts produced a strong suppression of hunger and negatively influenced subsequent food intake in a preload study. Several investigators have speculated that a major reason for the lack of predicted weight gain in long-term nut-supplemented diets is believed to be linked to dietary compensation [31,32]. Lastly, a loss of available energy has been observed due to fecal fat loss related to the incomplete mastication of nuts, [33,34] which in combination with food displacement would largely explain the protective effects of nuts on risk of overweight.

This current study's findings in the context of vegetables and fruits are in partial agreement with those from a recent review article of plant foods and childhood obesity [35] that reported the lack of a protective effect of fruit and vegetable consumption on the risk of childhood obesity. Reasons for mixed findings across plant-based food groups may include the wide variability in cooking and preparation methods of fruits and vegetables that contribute to differences in energy density and macronutrient composition, which modifies their effect on body weight.

A recent review on dairy intake and obesity in children and adolescents has addressed several reasons for inconsistent findings across observational studies, i.e. the problem of implausible dietary self-reporting, the uncertainty over whether the effect of dairy intake is independent of energy intake or other eating pattern variables, and, the lack of consensus on how to qualify or quantify dairy consumption [36]. We found a positive relationship and significant linear trend between the risk of being overweight and dairy consumption using a model that took into consideration the intake of the other food groups. Our study's results do not support the recent NHANES data that showed an inconsistent association between dairy intake and body fat indices among children and an association between suboptimal dairy intake and higher anthropometric measures of body fat among adolescents [37]. Our current findings are also in contrast to a review article on dairy and weight management that found that prospective and cross-sectional studies in children and adolescents showed either an inverse or neutral association between dairy intake and body weight [38]. It is possible that the aforementioned role of saturated fat may have contributed to our findings that dairy intake was associated with increased risk of overweight because there were fewer low-fat and non-fat dairy options available at grocery stores, fast food restaurants and school food programs in the 1980s.

The current study has several strengths worth noting. The study sample size was large, included both genders, and, featured a wide range of ages and dietary intakes. The non-quantitative FFQ was previously validated among a similar cohort of children and adolescents. The study population exhibited a wide range of intake for the foods; hence this large degree of interindividual variation increased the study's statistical power. The presence of foods that we classified as LNDF is less than the current levels consumed in our present day food environment.

In light of the relatively recent media focus on overweight among children and adolescents, the problem of underreporting of LNDF and over reporting of plant foods found among overweight children and caretakers in other studies [39-41] may have been attenuated. Previous studies have shown that social approval needs can cause a bias in reporting the frequency of food intake [42-44]. In one study, BMI was found to be a significant negative predictor of percentage of caloric intake from LNDF [45]. The tendency to underreport the consumption of LNDF foods among overweight children may have changed the direction of the association causing the normal weight children to report more LDNF consumption compared to their overweight counterparts.

In addition, we believe that the significant findings for a protective effect from LNDF are due to a combination of factors. First, reverse causation, i.e. parents with overweight children during the 1980's tightly restricted the consumption of LNDF in the home whereas parents of normal weight children had less reservation about keeping LNDF out of their child's reach or home environment. Secondly, lean children that are very physically active consume more food in general and, perhaps more LNDF.

Despite the fact that our data was collected in the 1980's when the prevalence of childhood obesity was much lower [2], the association between the plant-based food consumption and the likelihood of being lean is demonstrated here in our study. A similar finding was also observed in a more recent study of 215 adolescents attending 5 Adventist secondary schools where the association between vegetarian foods consumption and a lower measurement of BMI and waist circumference was reported [46]. Thus, despite the change in the food environment that may have occurred between the late 1980s and the present time, the association between plant-based foods consumption and leanness persists. This association was not only observed in the Adventist cohort but was also found in both children and adults in the non-Adventist cohorts [11,12,47,48]. The consistency in the association between plant-based consumption and lower BMI exists across time and continents suggesting that our findings can be applied to the general population in the current food environment.

This study is not without limitations. Due to lack of ethnicity data, we could not adjust for this covariate in the model. We could not adjust for total energy expenditure in the analysis because we did not have information on the daily number of TV viewing hours or time engaged in physical activity, which can be important contributors to the risk of overweight [49]. Also, due to the nature of the non-quantitative FFQ, we were unable to compute total energy intake and adjust for this in the analysis.

## Conclusions

In summary, a protective association for consumption of grains and nuts on risk of overweight was observed among children and adolescents living in Southern California in the 1980's in contrast to an increased risk of overweight found with dairy consumption. Grains, nuts and peanut butter are less costly than many animal-based foods and are enjoyed by most children and adolescents. However, plant-based foods have been traditionally underrepresented in school food programs. Our findings suggested that the intake of full fat dairy foods may be obesogenic, therefore it is important to continue to support public health guidelines that recommend the intake of reduced fat dairy products. In light of the current US administration's priority to curb child adolescent overweight, these topics will likely emerge for discussion and may fuel food policy revisions throughout our governmental programs. The 2005 Dietary Guidelines for Americans [50] and MyPyramid [51] currently emphasize the inclusion of a higher proportion of plant-based versus animal-based foods for optimal health, and, the preliminary report of the Advisory Committee for the Dietary Guidelines for Americans 2010 calls for Americans to shift toward a more nutrient-dense, plant-based diet to reduce the prevalence of overweight [52].

With surmounting evidence showing that vegetarian diet can provide a healthy alternative lifestyle at any age [53], we recommended plant-based foods as one sensible approach for the prevention of obesity in children and adolescents. Plant-based dietary patterns should be encouraged and promoted in the school food programs at the local and national levels.

Food policy at local, national and international levels is warranted to ensure that plant-based foods are affordable, accessible and a desirable selection among children and adolescents.

## Competing interests

The authors declare that they have no competing interests.

## Authors' contributions

VLM and JS were involved in the study design, data acquisition, data analysis and statistics. MW participated in study design and manuscript preparation. All authors have read and approved the final manuscript.
